# Integrated PTSD and Adherence Treatment for People with HIV: Main Findings of a Feasibility Pilot for Cognitive Processing Therapy-Lifesteps (CPT-L)

**DOI:** 10.21203/rs.3.rs-6567722/v1

**Published:** 2025-05-27

**Authors:** Cristina López, Nathaniel Baker, Stephanie Amaya, Erin Bisca, Tayler Wilson, Allison Ross Eckard, Angela Moreland, Patricia A. Resick, Steven A. Safren, Carla Kmett Danielson

**Affiliations:** Medical University of South Carolina; Medical University of South Carolina; Medical University of South Carolina; Medical University of South Carolina; Medical University of South Carolina; Medical University of South Carolina; Medical University of South Carolina; Duke University; University of Miami; Medical University of South Carolina

**Keywords:** People with HIV, PTSD, antiretroviral therapy, stigma, cognitive processing therapy

## Abstract

**Background:**

Despite high PTSD prevalence and related detrimental health outcomes for persons with HIV (PWH) there are no established integrated interventions addressing co-occurring HIV and PTSD. Negative reinforcement conceptual models posit that avoidant behavior (a hallmark symptom of PTSD) demonstrated by PWH with co-occurring PTSD can contribute to poor antiretroviral therapy (ART) adherence. However, research evaluating the impact of evidence-based treatment for PTSD among HIV positive populations on HIV outcomes is scarce. The Cognitive Processing Therapy (CPT) protocol is an evidence-based PTSD treatment that can address internalized stigma with tailored modifications and improve ART adherence and subsequent viral suppression through reduction of avoidant coping.

**Methods:**

This is the first pilot RCT to test feasibility of an integrated evidence-based PTSD treatment, cognitive processing therapy (CPT) with an adherence intervention (Life-steps) delivered in a Ryan White clinic to improve PTSD symptoms and HIV care. Participants were 41 adults with HIV, co-occurring PTSD, and areas of improvement in HIV care (e.g., missed appointments, less than 90% ART adherence, detectable viral load). Those meeting eligibility criteria were enrolled and randomized to the CPT-L intervention (n = 20) or the control condition of Life-steps only (n = 21). Outcome measures including PTSD symptoms, HIV stigma, quality of life, viral load, and engagement in care appointments were collected at baseline, post-intervention and at a 3-month follow-up visit.

**Results:**

Data from the feasibility trial demonstrated significantly greater reductions in PTSD symptoms (*Δ* = 11.55, *SE* = 4.32, *Cohen’s d* = 1.03, *p* = .01) and HIV stigma (*Δ* = 22.63, *SE* = 7.33, p = 0.006, *Cohen’s d* = 1.37) in participants randomized to CPT-L compared to Lifesteps-only. Preliminary data also indicate promising findings in HIV care (e.g., improved appointment rates, overperformance on viral load and ART adherence) for individuals in the CPT-L group.

**Conclusions:**

The research extends PTSD treatment approaches as a paradigm to integrate adherence counseling and improve ART adherence. This is an innovative use of established behavioral interventions and supports the U = U (Undetectable = Un-transmissible) campaign, potentially helping prevent the transmission of HIV through increasing viral suppression rates in a people living with HIV and PTSD.

The study was registered with clinicaltrials.gov, identifier NCT05275842.

## Introduction

As of 2022, there were an estimated 31,800 newly diagnosed people with HIV (PWH) in the United States, with 49% occurring in the Southern region of the country^1^. PWH experience a much greater exposure to traumatic events and prevalence of PTSD (30–74%)^2,3^ in comparison to the general population (3.9%)^4^, with interpersonal sexual and/or physical violence, childhood exposure to domestic violence and physical abuse, and seeing someone be seriously injured or killed as the most prevalent trauma exposures^5^. In addition, between 30 and 40% of PWH report their diagnosis, and impacts following the diagnosis, as their index trauma^6^.

Symptoms of PTSD, to include anxiety and depression^7^, as well as avoidance and distortion of cognitions, directly impact adherence to ART medication and retention in HIV care^8^. Conceptual models centering on the concept of negative reinforcement suggest that avoidant behavior tied to HIV status-related PTSD can contribute to poor ART adherence and to less success of viral suppression. Accordingly, cues associated with their ART medications can serve as a direct reminder of a traumatic event (e.g. a sexual assault) or can trigger negative cognitions associated with PTSD leading to hopelessness surrounding ART treatment benefits. Avoiding ART therefore is negatively reinforced. Furthermore, trauma exposure may impact cognitions surrounding trust, to include medical providers, and thus lead to delays in linkage and engagement in care. Suboptimal engagement and adherence to HIV care then can cause potential adverse outcomes, such as viral replication^9,10^, drug resistance^9,11^, disease progression^12^, and even death^13^ .

Stigmatization also can be both a direct barrier to adherence as well as a factor that influences additional barriers^14^. Three key ways in which stigma manifests itself is enacted (overt discrimination or rejection), anticipated (expectation of future stigmatization), and internalized^15^ (negative self-perceptions related to HIV. Individuals who identify with multiple minoritized and marginalized populations (i.e. women, LGBTQ+, racial and ethnic minorities), experience higher rates of intersectional stigma and non-adherence in HIV care^16,17^. Stigmatization can be experienced and anticipated by PWH in a variety of social and legal contexts, to include the workplace, school, residence^18^, and even healthcare clinics^19^. Other aspects associated with stigmatization that can impact access to care and further exacerbate health disparities within this population include socioeconomic status, health insurance, incarceration, and limited education^16,17,20–22^. Stigmatization within these various settings, along with avoidance behaviors, causes a decrease in social support, exacerbation of PTSD symptoms, elevated risk of transmission, reduced income, and declines in physical health^19,23^. In addition to potential effects of PTSD on HIV care outcomes, quality of life also suffers for PWH with co-occurring traumatic stress^24^. Studies have shown that individual’s quality of life is impacted and defined by socio-economic status, employment, social support, spirituality, physical and mental health, and overall satisfaction and happiness^24^. Although quality of life would be improved through active engagement in healthcare, factors such as poverty, substance use, domestic violence, depression, and HIV-related stigma influence PWH’s willingness to seek health related care^25^, thus further exacerbating low quality of life. In order to improve PWH’s overall quality of life mentally, socially, and physically, and in turn decrease the progression of the disease, it is imperative to target the unique impacts of stigmatization^26^ while also addressing the harmful effects of traumatic stress.

### Current Study

The current study fills a significant gap in the field by extending PTSD treatment approaches as a paradigm to reduce barriers to address both trauma symptoms and barriers to HIV care for PWH, including stigma. Cognitive Processing Therapy (CPT)^27^ is a gold standard PTSD treatment model that has strong evidence for reducing avoidance, substance use, and depression among adults with complex trauma.^28,29^ CPT also directly targets trauma-related self-blame and guilt^30,31^ two factors intimately tied to avoidant coping^32^. Furthermore, reductions in avoidance symptoms have been linked to increases in (non-HIV) medical regimen engagement, including medication adherence.^33^ Although effective interventions to address HIV medication adherence and PTSD are widely available, there are no established integrated approaches that address overlapping issues of co-occurring HIV with PTSD and related health disparities. Thus, this pilot randomized controlled trial is the first to examine the critical question of whether HIV outcomes can be improved among the large number of PWH with comorbid PTSD by treating PTSD symptoms to maximize benefits of health promotion interventions. We developed Cognitive Processing Therapy-Lifesteps (CPT-L), the integration of CPT and another empirically supported intervention that has been successfully used to improve PTSD- and HIV-related outcomes in this population (Life-steps for Medication Adherence)^34^ and examined feasibility of recruitment, retention, and patient outcomes. Compared to participants assigned to Life-steps alone, we hypothesized that participants randomly assigned to CPT-L would yield better outcomes for patients with respect to PTSD and stigma as well as medical outcomes of viral load, attended Ryan White appointments, and quality of life.

## Methods

### Participants

**Figure 1:**
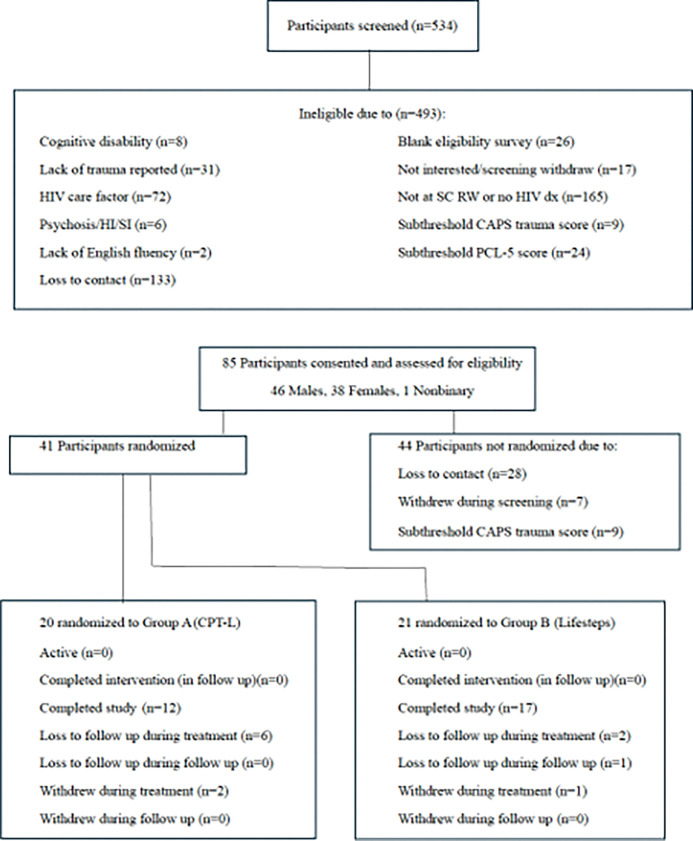
Consort Diagram

A total of 85 PWH diagnosed with PTSD were screened as eligible for study enrollment and 41 participants (48%) enrolled in study. Enrolled participants were randomized to receive the control condition of the adherence intervention alone (Life-steps, n = 21) or the intervention condition of CPT with integrated adherence components (CPT-L, n = 20; see Fig. 1 for CONSORT diagram).

Participants were recruited from surrounding Ryan White clinics within the state, including the Infectious Disease Department’s Ryan White Clinic at the academic medical center. Ryan White clinics are HRSA HIV/AIDS Bureau-funded programs providing a comprehensive system of HIV primary medical care, medications, and essential support service for low-income people with HIV. All protocols were approved by the Institutional Review Board at the affiliated university. Participants enrolled in the study met the following inclusion criteria: 1) age 18 or older; 2) enrolled in a Ryan White clinic; 3) reported 6 + PTSD symptoms across the four clusters (i.e., full PTSD clinical diagnostic threshold) on the CAPS-5 clinical interview^35^; 4) No changes in psychotropic medication within 4 weeks of study enrollment. 5) were able to speak, read, and write English; 6) Met at least one of the following HIV care criteria: a) diagnosed with HIV in last 3 months; b) detectable viral load in last 12 months; c) failed to show up for or missed 1 or more HIV care appointments in the past 12 months; d) Last HIV care visit was more than 6 months ago; e) self-reporting less than 90% ART adherence in the past 4 weeks. Exclusion criteria was very limited to ensure the most ecologically valid sample as possible. Exclusion criteria included: 1) evidence of significant cognitive impairment as assessed by the Montreal Cognitive Assessment Test (MoCA; in the severe range); 2) evidence of developmental delays, pervasive developmental disorder, or active suicidal or homicidal ideations; and 3) evidence of psychotic symptoms (e.g., active hallucinations, delusions, impaired thought processes; see [36] for more information about study design).

### Treatment Conditions

Participants assigned to Life-steps only (n = 21) as the control condition received the one session Life-steps intervention. Life-steps^34^ is evidence-based and uses cognitive behavioral strategies, problem-solving, and motivational interviewing techniques to promote adherence. Life-steps content includes psychoeducation on benefits of ART adherence, modification of maladaptive cognitions about taking ART, review of potential barriers to being adherent, and problem-solving techniques for problematic areas with adherence.

Participants assigned to the treatment condition, CPT-Life-steps (CPT-L) received an approach that integrates Cognitive Processing Therapy (CPT) for PTSD^27^ and Life-steps for medication adherence into a combined treatment protocol. CPT-L addresses PTSD-related avoidant coping and maladaptive thinking. In CPT-L, the patient details in two impact statements how the traumatic event (first statement) and HIV diagnosis (second statement) have impacted beliefs about self, others, and the world. The CPT-L therapist uses Socratic questions to explore unhelpful thoughts (e.g., self-blame) and modify maladaptive thinking (e.g., “I’m unlovable”). Upon developing skills to identify and address unhelpful thinking and internalized stigma, the patient restructures beliefs related to traumatic events and uses adaptive strategies to improve overall functioning and quality of life. CPT-L focuses on the original CPT themes of safety, trust, power, control, esteem, and intimacy, because these are domains affected by trauma and reflected in HIV stigma. CPT-L involves 12 sessions, 90 minutes twice a week.

### Procedures

Recruitment was achieved through several avenues: 1) clinicians and staff in partnering HIV care clinics referred patients with trauma backgrounds to the research team to discuss potential interest after sharing the referral link or a study flyer; 2) responses to trauma screening questions (Primary Care PTSD Screener for DSM-5^37^ included in several partnering clinics’ intake procedures of case managers; 3) providers and staff distributed REDCap^38^ eligibility survey to potential participants via e-mail as a way to identify potential eligible participants; 4) study flyers posted in surrounding clinics and via social media. The study’s purpose, methods, and compensation were discussed by research personnel with eligible participants, and if they were interested in participating, they completed an initial contact assessment. This initial assessment included completing a shortened version of the PTSD Checklist for DSM-5 (PCL-5)^39^, as well as the Montreal Cognitive Assessment (MoCA)^40^. If participants scored at least a 3 out of 5 on the shortened PCL-5, indicating possible PTSD, and scored within the mild or moderate range on the MoCA assessment (i.e. no indication of severe cognitive impairments), then the consent process was completed. Consented participants then completed the Clinician Administered PTSD Scale for DSM-5 (CAPS-5)^41^ to determine if they met criteria for PTSD. Participants who met criteria for PTSD were randomized by the PI using a REDCap generated assignments. A stratified randomized blocks procedure was used to assign participants in a 1:1 allocation while stratifying on potentially confounding variables. These stratification factors were detectible viral load (yes/no) at study entry and self-identified gender (as recommended by the Fenway Institute for SOGI [Sexual Orientation and Gender Identity]^42^ data collection. Baseline viral load stratification was chosen to assure balance as viral load was an acceptable surrogate for several of the proposed outcomes (viral load, ART adherence, visit attendance). Gender was chosen since women are twice as likely to develop PTSD than men.

Study assessment personnel were blinded to treatment condition, and assessments for both conditions took place in-person, via telehealth, or by phone for baseline, post-intervention (6 weeks) and 3- month follow ups (for more details regarding procedures see [28]). Individuals were compensated $50 for completing the baseline assessment, $40 for completing the first session, $60 for completing the post-intervention assessment and 20-minute exit interview, and $75 for completing the 3-month follow-up assessment for a combined possible total of $225.00. The study time frame for recruitment and follow-up was from May 2022 to August, 2024.

### Measures

**Demographics** were assessed during the baseline session and included sex, age, race, ethnicity, sexual orientation and gender identity (SOGI).

**Posttraumatic stress disorder** was assessed via self-report with the PTSD Checklist (PCL-5)^39^. The PCL-5 includes 20 items assessing PTSD symptoms consistent with the DSM-5 symptom criteria. The PCL-5 asks participants to rate their level of distress within the past month on 20 symptoms across four clusters, with ratings on a scale of 0 (*not at all*) to 4 (*extremely*). Higher scores indicate more severe PTSD, and scores range from 0 to 80. The PCL-5 is a validated measure and has revealed good validity and reliability with veterans^43^. A structured diagnostic interview was also completed. The Clinician-Administered PTSD Scale (CAPS-5) is a well-validated and widely used structured diagnostic interview for posttraumatic stress disorder (PTSD) that has demonstrated good internal consistency, interrater reliability, and test-retest reliability^35^. The CAPS-5^41^ includes items reflecting DSM-5 PTSD symptoms using a 5-point scale to assess PTSD diagnosis and symptom severity and frequency: 0 (absent), 1 (mild/subthreshold), 2 (moderate/threshold), 3 (severe/markedly elevated) and 4 (extreme/incapacitating).

HIV stigma was assessed using the Berger HIV-stigma scale^44^. The Berger HIV scale is a well validated measure, and examines four factors of HIV stigma: personalized stigma, disclosure concerns, negative self- image, and concerns with public attitudes towards PWH. The instrument comprises of 40-items, which responses rated on a 4-point Likert scale ranging from “strongly disagree” to “strongly agree”.

The validated World Health Organization Quality of Life HIV-related Scale (WHOQOL-HIV BREF^)45^ was used to measure HIV-related Quality of Life. It is a 26-item measure specifically designed to evaluate HIV-related quality of life (QOL) across 6 domains: 1) Physical health; 2) Psychological wellbeing; 3) Independence, 4) Social relationships; 5) Environment; and 6) Spiritual, religion, and personal beliefs. Responses to the items range from 1 (least favorable condition) to 5 (most favorable condition), with the domain scores, ranging from 4 to 20. A higher score would indicate a better QOL on the corresponding domain. It also includes two items on overall QOL and general health.

Self-reported ART adherence was assessed using the Measure of Adherence Scale (MAS). The MAS uses a three-item adherence scale, developed through thorough cognitive interviewing^46–48^. The three items included (1) an assessment of the number of days with missed ART doses in the preceding 30 days; (2) a scale rating of how good a job you did taking your medicines in the preceding 30 days and (3) a scale rating of how often you took your medicines the way you were supposed to in the preceding 30 days. The tool has demonstrated excellent internal consistency (α = 0.86) in diverse U.S. populations^46^.

Electronic medical records (EMR) and pharmacy records were reviewed for objective measures of participants’ viral load, ART adherence compliance, and attendance at Ryan White appointments. Baseline measures of viral load used medical record lab results as standard clinical practice within one year preceding baseline visit. Follow up viral load measures used blood work from EMR completed as part of a clinic visit within 3 months of study completion. HIV viral load less than 200 was considered the cutoff for virologic suppression. Estimates of ART adherence behavior were calculated from pill count photos provided by participants^49^ and medication possession ratio and percent of days covered by daily oral ART pulled from EMR and pharmacy dispensation records^50^. Adequacy of medication adherence was defined as taking greater than 80% of prescribed doses during the study period (yes/no). Appointment attendance with a prescribing HIV-care provider (e.g., excluding general specialists) was collected on a monthly interval via chart review/electronic medical record to document number of appointments made, number of no-shows, and number of cancellations within the time period.

### Study Sample and Randomization

Because the study is designed to demonstrate feasibility, sample size was determined based on pragmatic considerations, rather than through formal power analysis. The study aimed to randomize 60 PLWH to the CPT-L or Life-steps only condition (1:1), which is adequate to assess feasibility outcomes such as enrollment, attrition, retention, adherence to protocol and fidelity. Upon completion of study intake procedures, participants will be randomized to receive 3 months of CPT-L or Life-steps only using a stratified randomized blocks procedure to assign participants in a 1:1 allocation while stratifying on potentially confounding variables. These stratification factors are detectible viral load (yes/no) at study entry and self-identified gender (as recommended by the Fenway Institute for Sexual Orientation and Gender Identity data collection). Baseline viral load stratification was chosen to assure balance as viral load is an acceptable surrogate for several of the proposed outcomes (viral load, ART adherence, visit attendance). Gender was chosen since women are twice as likely to develop PTSD than men.

### Data Analytic Strategy

Study feasibility was determined by the overall completion rate of all participants at the end of study treatment and follow-up, as well as the proportion of study visits completed in each treatment group. Demographic and clinical characteristics were summarized for all randomized participants as well as stratified by study treatment assignment. Demographic and clinical characteristics measures at study baseline were summarized for the entire cohort as well as stratified by randomized treatment assignment. Between group comparisons were done using Wilcoxon Test for continuous characteristics and Chi-Square test for categorical characteristics (Fisher Exact test used when appropriate).

Changes in CAPS-5 scores were assessed at the end of study treatment (from baseline) as the primary marker of improvement in PTSD symptom severity. Changes in CAPS-5 total scores, PCL-5 total scores as well as the total number of CAPS-5 items rated as severe at the end of treatment were compared between treatment groups using linear regression models. Secondary longitudinal assessment of PTSD severity was done utilizing weekly PCL-5 assessments. Primary efficacy endpoints are derived from analysis models adjusted for baseline randomization stratification variables (gender and detectable HIV viral load) and pre-treatment trauma measurements. Model results are noted as the marginal group level mean change from baseline (standard error), group difference in change from baseline (standard error) and Cohen’s d (where appropriate). Pursuant to the protocol, we additionally examined the effects of sex, race and baseline viral load status (detectable) on changes in CAPS-5 outcomes. Although the study is not specifically powered to detect statistically significant treatment moderation by these factors, model interactions and stratification were assessed to provide effect sizes.

Secondarily, levels of HIV viral load, HIV Stigma and quality of life were assessed at study baseline and end of study treatment. End of study viral load suppression was assessed as the proportion of participants with less than 200 copies measured between end of study treatment and the 3-month follow-up visit. Stigma was measured using the Berger Stigma Scale and quality of life using the WHOQOL-bref for HIV patients. End of treatment viral load suppression was compared between groups using a fisher exact test while stigma and quality of life were compared using ANOVA models.

Objective measurement of medication adherence was measured twice during study participation using a combination of pill counts and pharmacy data^49,50^. End of treatment medication adherence data are summarized as the median proportion of doses measured at the final visit and compared between groups using a Wilcoxon Rank Sum test. Additionally, medication adherence was determined as taking at least 80% of prescribed doses and was compared between groups using a Chi-Square test. Self-reported HIV medication adherence was measured at study baseline and at end of study treatment using a validated 3-item Medication Adherence Scale (MAS)^47^. Items included the 30-day percentage of doses taken and 2 Likert measurements (6 level) examining the frequency of taking medication correctly and a rating of how good a job the participant did at taking medication as they were supposed to take it. The percentage of doses taken is compared between groups using Wilcoxon-Rank Sums test while Likert variables were binned as those that ‘almost always’ or ‘always’ took their medication with the correct frequency (vs. never, rarely, sometimes, usually) and those that rated their medication adherence as ‘very good’ or ‘excellent’ (vs. very poor, poor, fair, good) and compared between groups using Fisher Exact tests.

All statistical analysis were completed using SAS version 9.4 (SAS Institute Inc., Cary, NC, USA) and IBM SPSS Statistics (Version 30). Because this was a pilot study, statistical significance was assessed at an alpha level less than .05 and no corrections for multiple testing were conducted.

## Results

### Study, Retention, Feasibility and Flow

The current study screened 534 participants with 85 (15.9%) eligible for study enrollment. The primary ineligibility factors were ‘no HIV diagnosis/participation in a SC Ryan White clinic (n = 165, 33.5%), ‘loss of contact following screening/screening withdrawal’ (n = 150, 30.4%), missing HIV care criteria (n = 72, 14.6%), and ‘subthreshold trauma’ (n = 65, 13.2%). Of those considered eligible, 41 (48.2%) were randomized to receive the control condition (Life-steps only, n = 21) or the experimental condition (CPT-L, n = 20). Of the 41 randomized participants, 29 (71%) had end of treatment trauma data (Life-steps only, n = 17/21, 81% vs. CPT-L, n = 12/20, 60%, p = .14).

### Demographics and Clinical Characteristics

At baseline, study participants were 44.8 years old (SD = 12.3), 61% black (n = 25/41) and primarily male sex (63%, 26/41); **See** Table 1.

Participants randomized to receive CPT-L were slightly older (49.1 (11.8) vs. 40.8 (11.6) and more likely tobe white (40% (8/20) vs. 19% (4/21)) as compared to those randomized to Life-steps only. CAPS-5 totalscores (CPT-L: 33.5 (8.8) vs Life-steps only 37.0 (9.7); p = .26), CAPS-5 Severe item count scores (CPT-L12.9 (3.5) vs Life-steps only 14.1 (3.2); p = .28) and PCL total scores (CPT-L 36.8 (12.3) vs Life-steps only42.6 (11.3); p = .10) were similar between groups at baseline. In the baseline sample, 87.5% of participantshad suppressed viral load (n = 35/41; <200 copies).

### Primary Trauma Outcomes

**Figure 2:**
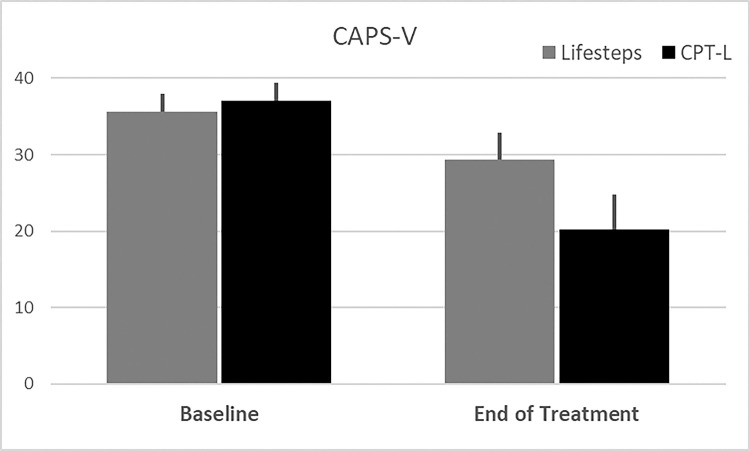
Baseline and end of treatment CAPS-5 scores. Note: Data Shown as Means and associated Standard Errors in participants with complete data across both measurements.

**Figure 3:**
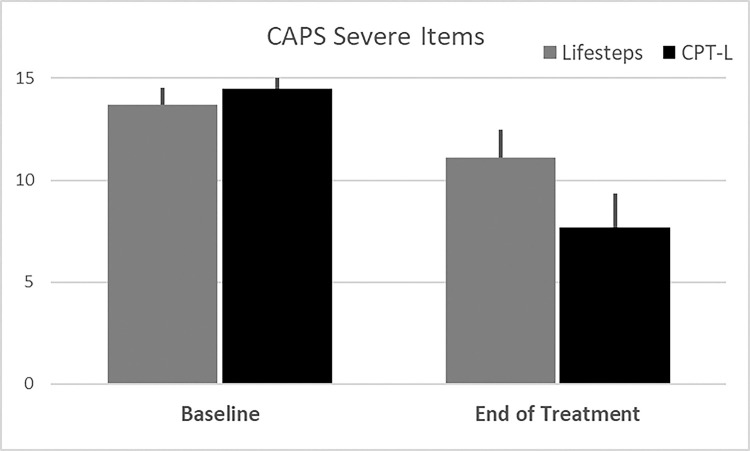
Baseline and end of treatment CAPS-5 severe item count. Note: Data Shown as Means and associated Standard Errors in participants with complete data across both measurements.

**Figure 4:**
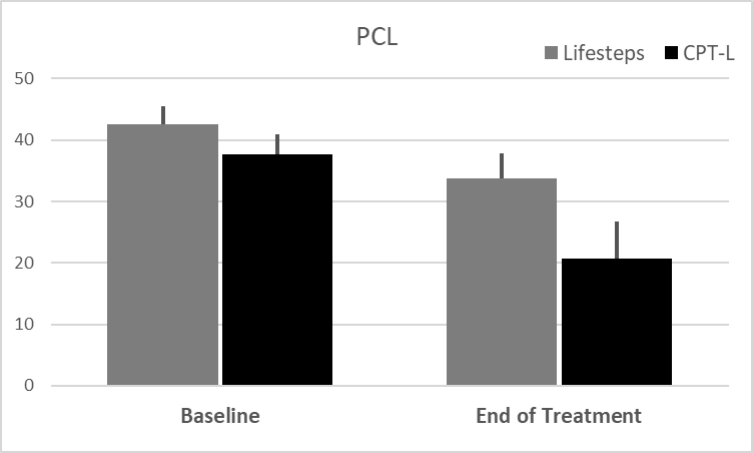
Baseline and end of treatment PCL-5 total score. Note: Data Shown as Means and associated Standard Errors in participants with complete data across both measurements.

Change from baseline to end of treatment CAPS-5 and PCL scores were assessed in all available participants and compared between groups. Univariable analysis of participants with available end of treatment study data indicated those randomized to receive CPT-L had a greater decrease in CAPS-5 total scores (CPT-L: Δ=−16.8 (3.2) vs. Life-steps only: Δ=−6.2 (2.7), p = .017); **See** Fig. 2) and the number of severe CAPS-5 items endorsed (CPT-L: Δ=−6.8 (1.4) vs. Life-steps only: Δ=−2.6 (1.1), p = .025); **See** Fig. 3) as compared to participants randomized to receive Life-steps only. Decreases in PCL scores at the end of treatment were numerically, but not statistically greater in the CPT-L participants as compared to the Life-steps only participants (Δ=−16.9 (5.1) vs. Δ=−8.9 (4.3), p = .24); **See** Fig. 4).

### Effect Modifiers: Sex, Race and Viral Load

The effect of CPT-L on CAPS-5 scores may vary by sex (treatment x sex interaction; p = .06) indicating that men had a differential response to treatment as compared to women. The treatment effect in men was positive and statistically significant (CPT-L=−21.8 (4.6) vs. Life-steps only=−3.7 (4.2), Δ = 14.6 (6.4), p = .03, Cohen’s d = 1.48) while non-significant in women (CPT-L=−7.2 (5.1) vs. Life-steps only=−6.1 (4.1), Δ = 1.1 (6.2), p = .87, Cohen’s d = 0.22). Although evidence that Black race had differential treatment efficacy (treatment x race interaction; p = .18), Black participants had significantly greater decreases in CAPS-5 scores following treatment as compared to all others (Black=−14.3 (3.1) vs. Other Races=−3.7 (3.5), Δ = 10.6 (4.1), p = .02, Cohen’s d = 0.62). Detectable HIV viral load at study entry did not modify treatment efficacy (treatment x HIV VL interaction; p = .25) nor was viral load predictive of treatment efficacy (undetectable=−10.3 (2.2) vs. detectible=−7.7 (4.6), Δ = 2.7 (5.1), p = .61, Cohen’s d = 0.39).

### Stigma

Changes in HIV-related stigma from baseline to six weeks were evaluated using Analysis of Covariance, controlling for the effects of baseline stigma, viral load, and gender. By six weeks, the CPT-L intervention produced greater improvements compared to the Lifesteps-only intervention across multiple facets of HIV-stigma (see Table 2). Specifically, the CPT-L intervention produced significantly more substantial reductions in total stigma (adjusted mean change = 30.16, SE = 5.55) versus Lifesteps-only (adjusted mean change = 7.53, SE = 4.29), F(1, 21) = 9.53, p = 0.006, Δ = 22.63 (7.33), Cohen’s d = 1.37. Similar patterns favoring CPT-L were found for concern with public image (10.31 vs. 3.10), F(1,23) = 7.16, Δ = 7.21(2.70), p = .014, Cohen’s d = 1.15, negative self-image (5.64 vs. 0.76), F(1,23) = 9.43, Δ = 4.88 (1.59), p = .005, Cohen’s d = 1.23, and disclosure concerns (4.29 vs. 0.79), F(1,24) = 6.07, Δ = 3.50 (1.41), p = .021, Cohen’s d = 0.96. Reductions in personalized stigma approached significance (7.78 vs. 2.17), F(1,23) = 3.79, Δ = 5.61 (2.88), p = .064, Cohen’s d = 0.80, with CPT-L showing a trend toward greater reductions. Baseline stigma was a significant covariate across all models.

### Medical Outcomes

Of the 41 randomized participants, 28 (68.3%) had post treatment viral load measured prior to the 3-month follow-up visit. Of those, 24 (85.7%) had suppressed viral load at the most recent measure (< 200 copies). In the CPT-L group 93.3% (14/15) had suppressed viral load while 76.9% (10/13) of those in the Life-steps only group had suppressed viral load (Fisher’s p = .31).

Quality of life was measured using the WHO-QOL Bref for HIV patients encompasses 6 quality of life subscales: Physical, psychological, independence, social, environmental and spiritual. At the close of study treatment, participants in the CPT-L group had statistically greater physical (CPT-L: 14.6, SE = 0.7 vs. Life-steps only: 12.7, SE = 0.6), p = .049), psychological (CPT-L: 13.3, SE = 0.7 vs. Life-steps only: 11.1, SE = 0.6), p = .024), environmental (CPT-L: 13.2, SE = 0.5 vs. Life-steps only: 11.4, SE = 0.4), p = .012), and spiritual (CPT-L: 14.9, SE = 0.6 vs. Life-steps only: 12.9, SE = 0.5), p = .026) quality of life as compared to participants in the Life-steps only group. Although statistically insignificant, participants in the CPT-L group had numerically greater Independence (CPT-L: 15.1, SE = 0.9 vs. Life-steps only: 12.9, SE = 0.7), p = .055) and social (CPT-L: 14.5, SE = 0.9 vs. Life-steps only: 12.8, SE = 0.7), p = .015) quality of life as compared to the Life-steps only condition.

Medication adherence was measured at the end of study treatment and summarized over the prior 30 days. In the study completers, the media medication adherence was 90.6%; 99.4% in the CPT-L group compared to 73.1% in the Life-steps only group (Wilcoxon Rank Sum P = .19). Further, 15 of the 29 completers reported medication compliance (≥ 80% of doses taken) with 66.7% (8/12) in the CPT-L group and 41.2% (7/17) in the Life-steps only group (χ^2^_1_ = 1.8; p = .18). In addition to objective measurements of medication compliance, the Medication Adherence Scale was used to collect self-report ratings of medication compliance. At the close of study treatment, 100% of reporting participants (13/13) in the CPT-L group noted that they took their medication ‘always’ or ‘almost always’ as prescribed compared to 81.3% (13/16) of participants in the Life-steps only group (Fisher’s p = .23). Similarly, 92.3% (12/13) of participants in the CPT-L group rated their medication adherence as ‘excellent’ or ‘very good’ as compared to 75% (12/16) or participants in the Life-steps only group (Fisher’s p = .34).

Ryan White Clinic attendance was measured during study treatment and through the 3-month follow-up visit. Out of the 41 randomized participants, 35 had HIV clinic appointments scheduled between study randomization and follow-up. Out of 104 scheduled clinic visits, 62 (59.6%) were missed; 25 of 47 (53.2%) visits were missed in the CPT-L group and 37 of 57 (64.9%) in the Life-steps only group (χ^2^_1_ = 0.7; p = .41). Additionally, 17 of the 41 (41.5%) randomized participants had at least 1 hospitalization during treatment or follow-up; 7 of 20 (35.0%) in the CPT-L group and 10 of the 20 (50.0%) in the Life-steps only group (χ^2^_1_ = 1.1; p = .29).

## Discussion

The current study conducted a pilot RCT to assess preliminary efficacy, acceptability, and feasibility for CPT-L, an integrated intervention to decrease PTSD symptoms and improve HIV care outcomes among PWH with co-occurring traumatic stress. Using PTSD treatment approaches as a new paradigm to improve HIV care, CPT-L incorporated a novel theoretical approach to address overlapping factors (e.g., avoidant coping) that affect onset and maintenance of PTSD symptoms as well as HIV-related stigma. PTSD and stigma are both well documented in the literature as common barriers to HIV adherence and viral suppression, yet evidence-based interventions have not tested approaches to simultaneously address both of these deleterious contributors to poor HIV care management. CPT-L is both innovative and necessary given the disproportionate impact of traumatic stress and stigma on PWH and disparities along the HIV treatment cascade that are exacerbated by PTSD.

In a sample intended for feasibility, participants receiving CPT-L demonstrated strong and statistically significant decreases in PTSD symptoms compared to participants randomly assigned to the single session Life-steps only intervention. Consistent with hypotheses, CPT-L also resulted in more statistically significant decreases in HIV internalized stigma compared to individuals receiving Life-steps only. Given the impact of CPT-L on PTSD and stigma, integration of PTSD treatments on HIV and other stigmatized chronic disease models merits further investigation. Although CPT has been enhanced to target alcohol use and high risk sexual behavior to prevent HIV among American Indian women^51^, CPT, nor other gold-standard, empirically supported individual treatments (e.g., Prolonged Exposure/PE)^52,53^, have been adapted for PWH populations, nor have they been evaluated as an efficacious approach to reduce problematic HIV outcomes. Findings from this feasibility study highlight the critical need to examine the utility of evidence-based treatments for PTSD among PWH and co-occurring PTSD to improve HIV outcomes.

As demonstrated with the main effects on reducing HIV-related stigma, CPT may be particularly beneficial for PWH due to the focus on unhelpful cognitions, or “stuck points,” in the form of assimilation (beliefs related to self-blame) and overaccommodation (extreme beliefs related to self and world)^54^. The flexibility of CPT to focus on unhelpful thoughts and thinking patterns may allow patients to address both trauma- and HIV-related stigma cognitions in treatment. In line with the goal of the Undetectable = Untransmittable (U = U) initiative to end HIV-related stigma, the integration of stigma and PTSD interventions can have broad implications not only from a public health standpoint, but also for the self-esteem of individuals by reducing internalized stigma associated with HIV.

In addition to reduced mental health outcomes, this feasibility study collected data on viral load suppression, ART adherence, and attendance at Ryan White care appointments as HIV care outcomes that can be subsequently tested as CPT-L treatment effects. A descriptive review of post treatment viral load data from this pilot RCT demonstrated that 93.3% of participants in the CPT-L condition had suppressed viral load while 76.9% of those in the Life-steps only condition had suppressed viral load. Although not statistically different with this sample size, descriptive data in both objective and self-report measures of ART adherence also indicated greater medication adherence in CPT-L (67%) compared to participants in Life-steps only group (41%). Although findings should be interpreted with caution, these results are notable because both conditions received the Life-steps only adherence intervention to achieve viral suppression. Taken together, these preliminary findings of effects of CPT-L on improved HIV care outcomes show promise and warrant continued evaluation. Similarly, preliminary results of post-treatment attendance at HIV care appointments showed encouraging patterns of improvement for participants in the CPT-L condition: a larger proportion of participants in the Life-steps only group (65%) “No-Showed” at least one appointment with their ART prescribing provider, compared to (53%) of those in the CPT-L group. Given that adherence to primary care appointments at Ryan White facilities has been strongly associated with achieving durable viral suppression^55^, reduced rates of no-show appointments serves as a promising marker for the ability of CPT-L to move the HIV care needle. These favorable findings on three common metrics of HIV care encourage us to evaluate CPT-L with a larger sample that is adequately powered to assess the true efficacy of CPT-L on viral suppression, ART adherence, and retention in Ryan White appointments. These HIV care study findings, coupled with high acceptability of CPT-L and enthusiastic qualitative feedback from participants and community stakeholders^56^, highlight the need to examine effects of CPT-L on HIV care on a larger scale.

Our results from the CPT-L pilot RCT extend recent efforts in examination of interventions that address traumatic stress and that have also begun to signal improvements in HIV care. For example, the Improving AIDS Care after Trauma (ImpACT) program, a hybrid individual and group-based coping intervention for women with sexual abuse histories living with HIV in South Africa, reported reductions in avoidance and arousal related to PTSD and greater increases in ART adherence motivation compared to control participants^57,58^. Domestically, Dale and colleagues recently shared that an integrated empowerment intervention (STEP-AD) reduced PTSD symptoms and improved HIV viral load among Black women^59^. Our study builds upon these findings by adapting already established evidence-based PTSD protocols and integrating tailored content for PWH identifying with a wider array of ethnic identities, thus providing broader population impact (i.e., both STEP-AD and ImpACT interventions are exclusively for Women with HIV). Interestingly, while CPT-L led to significant reductions across both men and women, there were significantly greater improvements in reductions of PTSD symptoms in male participants. Given that 66.3% of HIV incidence within the United States in 2022 was comprised of Black men^60^, our results reaffirm the importance of identifying psychosocial interventions that can reduce PTSD-related symptoms and move the HIV care needle among broad segments of the populations affected by HIV. CPT-L has the potential to improve HIV-related outcomes for individuals from several minoritized racial and sexual backgrounds that have experienced traumatic stress affecting their HIV care management. Larger scale dissemination of CPT-L would be feasible on a national scale using the Ryan White network^61^.

While CPT-L has several novel and promising findings related to improvement of mental health and HIV care, a few limitations should be noted. First, as a feasibility study, our sample size of 41 participants limited our power to detect significant findings, especially with respect to HIV care outcomes. Second, in addition to electronic pharmacy records and self-report of ART adherence, use of electronic devices (e.g., Wisepill, MEMScaps) to capture real time adherence would help compare the results of CPT-L to other adherence interventions. Other indices of HIV care engagement could also be collected, such as the Index of Engagement in HIV care^62^ to help identify participants who would most likely benefit from an adherence intervention. Finally, improved retention would increase the data available to test the efficacy of CPT-L. CPT-L participants had end of trauma treatment data for 55% of the sample. While these numbers corroborate completion rates of other studies with PTSD treatment in populations that experience increased levels of traumatic stress (e.g., veterans, 30–50% treatment dropout rates)^63,64,^ studies delivering CPT sessions more than once a week have reported higher completion rates (e.g., 87%)^65^. The CPT-L protocol was encouraged to be completed twice a week, but flexibility was potentially overly permitted; future studies will include emphasis on twice a week appointments at the consent process so that participants are more likely to engage on a more intensive (and potentially more successful) schedule. Subsequent studies evaluating CPT-L should also include examination of potential mechanisms responsible for the successful reductions in stigma and improved HIV care models. Avoidant coping, a shared symptom of PTSD and HIV stigma, has been effectively reduced by CPT in relation to PTSD symptoms^30–32^ and may serve as a putative mediator of change.

## Conclusions

In conclusion, this pilot RCT of CPT-L among PWH with clinically significant diagnoses of PTSD demonstrated feasibility and preliminary efficacy with PWH in CPT-L compared to the participants in the Life-steps only enhanced control condition. PWH with PTSD continue to show worse outcomes related to HIV care and CPT-L may serve as a novel approach to address shared pathways leading to HIV related non-adherence (e.g., skipped medications, lack of trust with new medical provider). Evaluating evidence-based PTSD treatments to target specific needs of hard-to-engage populations can significantly impact public health by improving patient outcomes, engagement, and medication adherence across pathologies (e.g., viral suppression in PWH) and health promotion efforts. Future studies of CPT-L are needed to test the full efficacy on PTSD and HIV outcomes as well as potential mediators of this promising integrated intervention.

## Figures and Tables

**Table 1:** Baseline Demographic and Clinical Characteristics.

	Overall N = 41	CPT-L N = 20	Life-steps Only N = 21
Age,years	44.8 (12.3)	49.1 (11.8)	40.8 (11.6)
**Race**			
Black	61.0% (25)	55.0% (11)	66.7% (14)
White	29.3% (12)	40.0% (8)	19.1% (4)
MTO/Other	9.8% (4)	5.0% (1)	14.3% (3)
**Sex**			
Male	63.4% (26)	65.0% (13)	61.9% (13)
Female	36.6% (15)	35.0% (7)	38.1% (8)
Gender Identity			
Male	61.0% (25)	65.0% (13)	57.4% (12)
Female	36.6% (15)	35.0% (7)	38.1% (8)
Transgender Male	0.0% (0)	0.0% (0)	0.0% (0)
Transgender Female	0.0% (0)	0.0% (0)	0.0% (0)
Genderqueer	0.0% (0)	0.0% (0)	0.0% (0)
Additional Gender Category	2.4% (1)	0.0% (0)	4.8% (1)
**Baseline Measures of Study Outcomes**			
CAPS-5 Total Score	35.3 (9.3)	33.5 (8.8)	37.0 (9.7)
CAPS-5 Severe Item Count	13.5 (3.4)	12.9 (3.5)	14.1 (3.2)
PCL-5 Total Score	39.7 (12.0)	36.8 (12.3)	42.6 (11.3)
Suppressed Viral Load (< 200 copies)*	87.5% (35)	85.0% (17)	90.0% (18)
WHOQOL HIV - Bref			
Physical	12.3 (2.5)	12.9 (2.4)	11.8 (2.6)
Psychological	11.6 (2.7)	11.6 (2.8)	11.6 (2.6)
Independence	12.9 (3.2)	13.4 (3.3)	12.4 (3.1)
Social	12.0 (3.4)	12.5 (3.3)	11.4 (3.5)
Environment	11.6 (2.0)	11.8 (2.2)	11.4 (1.8)
Spiritual/Religious	12.8 (3.2)	13.4 (2.5)	12.2 (3.6)
**Berger Stigma Scale (M, SE)**			
Personalized Stigma	45.59 (1.86)	43.55 (2.12)	47.74 (3.07)
Disclosure Concerns	30.98 (1.03)	28.89 (1.54)	32.86 (1.28)
Negative Self-Image	33.87 (1.52)	29.83 (1.86)	37.33 (2.08)
Concerns with Public Attitudes about PWH	52.61 (1.76)	49.25 (1.99)	55.81 (2.73)
Overall Stigma	147.39 (34.79)	171.53 (39.92)	152.78 (30.91)

**Table 2:** Comparison of CPT-L and Lifesteps-only on HIV Stigma Reduction

	Lifesteps-only		CPT-L						
Measure	Mean (SD)	Adj Mean (SE)	Mean (SD)	Adj Mean (SE)	Mean Difference (SE)	*F*	*p*	Partial *η^2^*	Cohen’s *d*
Total Stigma	9.38 (19.53)	7.53 (4.29)	27.20 (12.67)	30.16 (5.55)	22.63 (7.33)	9.53	.006	.312	1.37
Personalized Stigma	3.31 (8.70)	2.17 (1.82)	6.25 (6.17)	7.78 (2.12)	5.61 (2.88)	3.79	.064	.141	0.80
Disclosure Concerns	1.24 (3.67)	0.79 (0.85)	3.67 (2.96)	4.29 (1.04)	3.50 (1.41)	6.07	.021	.202	0.96
Negative Self-Image	1.76 (3.90)	0.76 (0.92)	4.09 (4.81)	5.64 (1.18)	4.88 (1.59)	9.43	.005	.291	1.23
Concern with Public Attitudes	4.71 (7.27)	3.10 (1.60)	7.82 (8.66)	10.31 (2.03)	7.21 (2.70)	7.16	.014	.237	1.15

**Note**: Adjusted means account for covariates (corresponding baseline stigma, baseline viral load, and gender). Cohen’s d values were calculated using the formula d = 2√F/√df _error_. Effect size interpretations based on Cohen’s conventions: Small (d = 0.2), Medium (d = 0.5), Large (d ≥ 0.8).

## Data Availability

The dataset used and analyzed during the current study is available from the corresponding author on reasonable request.

## Abbreviations

ART: Anti-Retroviral Therapy
CAPS-5: Clinician Administered PTSD Scale for DSM-5
CPT: Cognitive Processing Therapy
CPT-L: Cognitive Processing Therapy and Life-steps
EMR: Electronic Medical Records
MAS: Medication Adherence Scale
MoCA: Montreal Cognitive Assessment
PCL-5: PTSD Checklist
PWH: Persons with HIV
QOL: Quality of Life
SOGI: Sexual Orientation and Gender Identity
U = U: Undetectable = Untransmissible
